# Potential mechanisms underlying sleep disturbance in young people with borderline personality disorder features: an exploratory study

**DOI:** 10.1186/s40479-022-00180-2

**Published:** 2022-03-10

**Authors:** Claire A. Jenkins, Katherine N. Thompson, Christian L. Nicholas, Jessica A. Hartmann, Andrew M. Chanen

**Affiliations:** 1 Melbourne School of Psychological Sciences, The University of Melbourne, Melbourne, Australia; 2 Orygen, Melbourne, Australia; 3 Centre for Youth Mental Health, The University of Melbourne, Melbourne, Australia; 4 Institute for Breathing and Sleep, Melbourne, Australia

**Keywords:** Borderline personality disorder, Sleep, Psychiatry, Emotion regulation, Actigraphy

## Abstract

**Background:**

Sleep disturbance is common among young people (15–25 years) with features of borderline personality disorder (BPD). However, the mechanisms underlying sleep disturbance in BPD remain unknown. Understanding these underlying processes is essential to guide the development of sleep-improvement interventions and to optimise their efficacy through identifying beneficial treatment targets. This exploratory study aimed to investigate potential underlying mechanisms to inform future hypotheses, research development, and provide insight into potential treatment targets to improve sleep in young people with BPD. This study explored the indirect roles of emotion regulation difficulties, depression, anxiety and stress in the relationship between BPD features and sleep disturbance in young people.

**Methods:**

Sleep was measured subjectively (self-report questionnaires) and objectively (10 days wrist actigraphy) in 40 young people with BPD features and 38 healthy young people. Participants also completed the Difficulties in Emotion Regulation Scale and the Depression, Anxiety and Stress Scale.

**Results:**

Mediation analyses revealed that impulse control difficulties, limited emotion regulation strategies and anxiety indirectly affected the relationship between group (BPD vs. healthy) and subjective sleep disturbance in young people. Lack of emotional awareness and anxiety contributed to associations between group and objectively longer time in bed and bedtime variability, respectively.

**Conclusions:**

These preliminary findings suggest that targeting emotional dysregulation (impulse control, strategies, emotional awareness) and anxiety might be beneficial for improving sleep in this population.

**Supplementary Information:**

The online version contains supplementary material available at 10.1186/s40479-022-00180-2.

## Background

Sleep problems are common in young people (aged 15–25 years) with features of borderline personality disorder (BPD), a severe mental disorder characterised by unstable interpersonal relationships, emotions and self-image, and impulsive behaviours [3, 41, 42]. Compared with healthy young people, individuals with BPD features report poorer subjective sleep quality and display objectively longer, later and more irregular sleep timing [38, 41, 40, 75]. However, the mechanisms underlying the relationship between BPD features and sleep problems remain largely unknown. Understanding these underlying processes is essential to guide the development of sleep-improvement interventions and to optimise their efficacy through identifying beneficial treatment targets. This study focussed on emotion regulation difficulties, depression, anxiety and stress, each of which are common in young people with BPD and independently associated with sleep disturbance.

### Emotion regulation difficulties

Emotional dysregulation, a defining feature of BPD [19, 26], is bidirectionally associated with sleep disturbance [27, 61]. That is, emotion regulation difficulties disturb sleep, through heightened arousal and negative affect, while sleep problems exacerbate emotional dysregulation [6, 21, 27, 34, 63]. Young people, whose emotion regulation skills are still developing, might be particularly susceptible to the emotional effects of poor sleep [63]. Nightmares, which are common in individuals with BPD, can also exacerbate daytime emotional dysregulation [68] and might even increase the risk of developing BPD by exacerbating trauma responses [79]. Therefore, understanding the relationships between BPD features, emotional dysregulation, and sleep problems during this developmental period is crucial.

Two studies have explored the role of emotional dysregulation in sleep disturbance in young people with BPD features [29, 75]. Grove et al. [29] found that global emotion regulation difficulties contributed to the relationship between BPD features and subjective sleep disturbance in university undergraduates. Subsequent research in a clinical sample, however, observed no effect of emotion regulation difficulties in this relationship and highlighted the need for additional research that includes objective sleep measures [75].

Grove et al. [29] also explored specific aspects of emotional dysregulation. Only lack of emotional awareness and clarity, and limited access to emotion regulation strategies contributed to the relationship between BPD features and subjective sleep disturbance [29]. These findings warrant replication and clarification in a clinical sample as it remains unclear why only these aspects of emotional dysregulation were found to contribute to sleep disturbance in BPD [29].

### Depression, anxiety and stress

The null findings from Wall et al. [75] suggest that other factors related to emotional dysregulation, such as depression, anxiety, or stress, might underlie sleep problems in this population. Depression, anxiety, and stress commonly co-occur with BPD in young people [12, 78] and are independently associated with sleep disturbance [2, 57, 64]. Although sleep problems in BPD cannot be solely attributed to co-occurring depression, the two disorders share similar sleep profiles [30, 59, 79]. As such, depressive symptoms might still contribute to sleep problems in individuals with BPD features, albeit indirectly.

Pre-sleep arousal is a key contributor to sleep disturbance, and specifically exacerbates subjective sleep problems [33, 71]. Yet, anxiety and stress have been largely neglected as potential underlying mechanisms in the field of sleep and BPD [79]. Pre-sleep arousal might be further exacerbated by nightmares in individuals with BPD features, who report heightened dream anxiety and fear of falling asleep due to anticipating a nightmare [69]. Anxiety and stress-related arousal, combined with heightened baseline arousal in BPD [48, 68], might contribute to sleep disturbance in this population.

To our knowledge, no studies have explored the roles of depression, anxiety and stress alongside emotional dysregulation, which is crucial given that these factors are interrelated [24, 45].

### The current study

This exploratory study aimed to investigate potential underlying mechanisms to inform future hypotheses and research development. It explored the roles of emotional dysregulation, depression, anxiety and stress in the sleep profile of young people with BPD features. It aimed to extend previous research by using a combination of subjective and objective (actigraphy) sleep measures, by investigating emotional dysregulation, and by exploring the comparative strengths of these potential mechanisms.

## Methods

### Participants

Participants were 78 young people (aged 15–25 years), 40 with BPD features (36 female, *M*_*age*_ = 19.77, *SD*_*age*_ = 2.51) and 38 healthy young people (34 female, *M*_*age*_ = 20.06, *SD*_*age*_ = 2.52). This age range represents a developmentally coherent group [11, 14], and a previous study using the same sample revealed no significant differences between adolescents (aged 15–17 years) and young adults (aged 18–25 years) with BPD features [42]. Within the BPD group, eleven (27.5%) participants had sub-threshold BPD (3–4 features) and 24 (60%) were taking prescribed psychotropic medication. By design, t here were no significant group differences in age or socioeconomic status, and sex distributions were comparable across groups to help eliminate any potential confounding effects of these factors on sleep. See [42] for detailed sample characteristics.

Participants with BPD features were recruited through the Helping Young People Early (HYPE) Program [13] at Orygen, the government-funded specialist mental health service for young people (aged 15–25 years) residing in western metropolitan Melbourne, Australia. Participants had three or more features of BPD, assessed using the Structured Clinical Interview for the Diagnostic and Statistical Manual of Mental Disorders, Fifth Edition (DSM-5) Personality Disorders (SCID-5-PD). Individuals with 3–4 BPD features were included, given the clinical significance and comparable functional impairment of individuals with sub-threshold BPD to those who meet full diagnostic criteria [47, 72, 80]. Exclusion criteria included a current DSM-5 diagnosis of schizophrenia spectrum disorder or bipolar I disorder, or a diagnosis of sleep apnea or a score above 0.5 on the multivariable apnoea risk index (MAPI [51];).

Healthy participants were recruited via word of mouth and social media (Facebook and Instagram). They had zero SCID-5-PD assessed BPD features, had never received any mental health treatment or been diagnosed with any mental or sleep disorders, and had a score below 0.5 on the MAPI.

The current study adhered to the Declaration of Helsinki and was approved by Melbourne Health Human Research Ethics Committee (#2017.153).

### Measures

#### Predictor variable

Group status was used as the predictor variable wherein 0 indicated a healthy participant and 1 indicated a participant with BPD features. Diagnoses were assessed using the Structured Clinical Interview for DSM-5, Research Version (SCID-5-RV: [22]) and SCID-5-PD [23]. Healthy individuals did not complete the SCID-5-RV and only completed the BPD module of the SCID-5-PD. Healthy participants were instead asked to report any mental disorder diagnoses (past or present) and any history of help-seeking (eg. seeing a school counsellor or psychologist).

#### Mediating variables

##### Difficulties in emotion regulation (DERS)

The DERS is a 36-item self-report questionnaire with high internal consistency (⍺ = 0.93) and good test-retest reliability (ρ_I_ = 88) [28]. Items are rated on a scale of 1 (“almost never [0–10%]”) to 5 (“almost always [91–100%]”), where higher scores represent greater difficulties in emotion regulation. The DERS yields a global score and six subscales: nonacceptance, goals, impulse, awareness, strategies, and clarity.

##### Depression anxiety and stress scales (DASS) 21

The DASS-21 is a 21-item self-report measure [49]. Each item is rated from 0 (never) to 3 (almost always), where higher scores indicate greater distress. The DASS includes three subscales, each with good internal consistency: depression (⍺ = 0.91), anxiety (⍺ = 0.84), and stress **(**⍺ = 0.90).

#### Outcome variables

##### Pittsburgh sleep quality index (PSQI)

The PSQI is a 9-item self-report measure of global sleep quality over the previous month [8], with good internal consistency (α = 0.87 [4]). It has seven component scores: sleep quality, sleep latency, sleep duration, sleep efficiency, sleep disturbances, use of sleeping medications, and daytime dysfunction. Each component is scored from 0 (no difficulty) to 3 (severe difficulty). A global score is calculated by summing the seven components, where higher scores represent poorer sleep quality. The global score was used in the current study.

##### Insomnia severity index (ISI)

The ISI is a 7-item measure of an individual’s clinical level of insomnia over the past 2 weeks [54]. The ISI has high internal consistency, α = 0.90 [55]. The index addresses difficulties initiating and maintaining sleep, sleep-related satisfaction and worry/distress, and the functional impact of insomnia symptoms. Each item is rated on a scale from 0 to 4 and a total score is calculated by summing all ratings, where higher scores reflected more severe insomnia.

##### Epworth sleepiness scale (ESS)

The ESS is an 8-item measure of an individual’s general level of daytime sleepiness [43], with good internal consistency, α = 0.88 [44]. Individuals rate their chances of dozing off or falling asleep while engaged in eight different activities on a scale of 0 (never) to 3 (high chance). A total sleepiness score is calculated by summing the eight ratings.

##### Consensus sleep diary

This sleep diary is a widely used and validated 15-item self-report measure [9, 50]. The diary assesses sleep timing, quality and behavioural factors that might affect sleep (eg. caffeine and alcohol intake, napping, medication use). Only bed and rise times from the diary were used in the current study, to inform the sleep period for actigraphy analyses.

##### Actigraphy

A GENEActiv® Original (ActivInsights Ltd., Cambridge, UK) triaxial accelerometry-based activity monitor was used to provide activity counts (per minute), configured with a sampling frequency of 50 Hz. GENEActiv data stored in 60-s epochs were downloaded for analysis using GENEActiv PC software (Activinsights; version 3.2). Data for all participants were visually checked against sleep diary data to confirm bed and rise times. The Philips Respironics algorithm [58] with a wake threshold of 115 was used to calculate sleep parameters outlined in Table 1. Research supports use of this algorithm and device-specific wake threshold (see Jenkins, Tiley et al., [40] for more information).

**Table 1:** Objective Sleep Parameter Definitions

Sleep Parameter	Definition
Bedtime	Time at which an individual started trying to fall asleep. This was pre-set based on sleep diary data and visual inspection of actigraphy data
Rise time	Time of final awakening, after which the individual did not attempt to fall back asleep. This was pre-set based on sleep diary data and visual inspection of actigraphy data
Time in Bed	The total number of minutes elapsed between bedtime and rise time
Total Sleep Time (TST)	The total number of minutes scored as sleep
Sleep Efficiency (SE)	Total sleep time divided by time in bed, expressed as a percentage
Sleep Onset Latency (SOL)	The time elapsed between bedtime and the start of the sleep period (the first epoch of the first section of 5 consecutive minutes scored as sleep)
Wake After Sleep Onset (WASO)	Time spent awake during time in bed after the start of the sleep period
Total Sleep Time Variability	Coefficient of variation (standard deviation/mean) for total sleep time within each participant
Time in Bed Variability	Coefficient of variation for time in bed within each participant
Bedtime Variability	Individual standard deviation for bedtime across the 10-day period
Rise time Variability	Individual standard deviation for rise time across the 10-day period

*Note*. Coefficient of variation (CV) has been used previously in sleep research in young people with mental illnesses ([38, 56]). CV can only be calculated with true ratio data. Hence, intra-individual variability in bed and rise times were assessed using individual standard deviations

#### Procedure

Informed consent was obtained from all participants and from a parent or guardian for participants aged under 18 years. Following screening for inclusion/exclusion criteria, participants completed the psychopathology interviews (SCID-5-RV; SCID-5-PD) and self-report questionnaires. Participants were provided with a GENEActiv device which they were instructed to wear continuously on their non-dominant wrist for the following 10 days. Participants were also asked to complete the sleep diary each day while wearing the device. Data collection took place during the educational term/semester (not during vacation or break periods) for individuals engaged in education at the time of participation, to ensure that any observed sleep disturbances were not attributable to being on break/off schedule.

#### Data analysis

Bed and rise times were converted to minutes for analysis. Midnight was treated as zero, such that times before midnight were converted to negative values (eg. 11:30 pm = −30mins) and times after midnight were converted to positive values (eg. 3:00 am = 180mins).

All aims were tested via mediation analyses, using non-parametric bootstrapping in PROCESS (Model 4, [36]). PROCESS is a widely used SPSS macro developed to conduct mediation and moderation analyses [36]. Indirect effects were considered significant if 95% bias-corrected confidence intervals (BCa CI), bootstrapped on 5000 resamples, did not contain zero. Across all models, the independent variable was a binary group variable, where 0 indicated a healthy participant and 1 indicated a participant with BPD features. Sleep was the dependent variable and separate mediation models were analysed for each sleep parameter (PSQI, ISI, ESS, actigraphy variables). Correlations between group, mediating and outcome variables were assessed prior to mediation analyses.

**Fig. 1:**
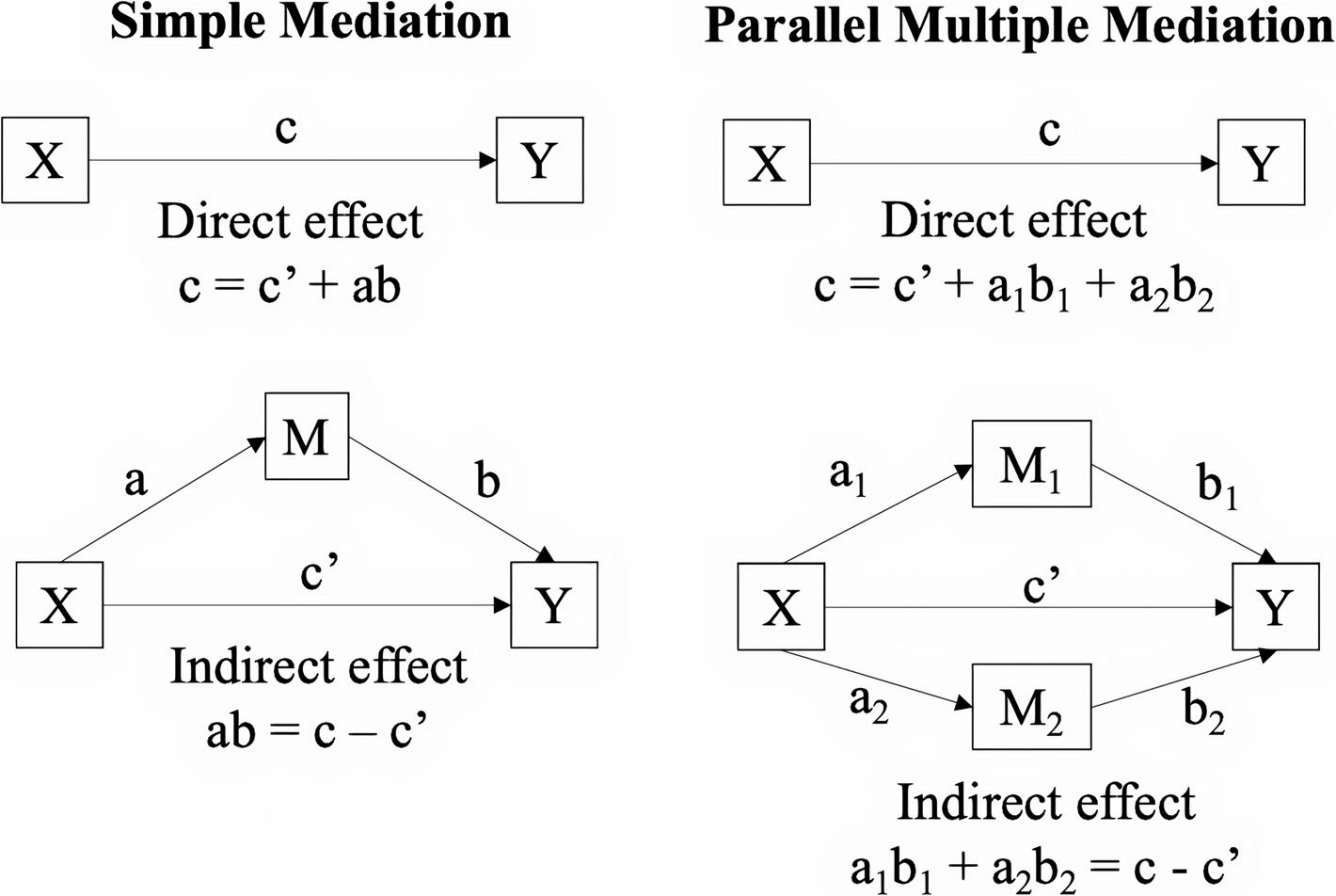
Conceptual Diagrams of Simple and Parallel Mediation Models. *Note*. X = independent variable. Y = dependent variable. M = mediating variable

Separate simple mediation analyses examined the indirect effect of each DERS subscales as well as DERS total score in the relationship between BPD and sleep disturbance. If significant indirect effects were observed for more than one DERS subscale, a parallel multiple mediation model was analysed (including all six DERS subscales as parallel mediators) to determine which had the strongest effect. Parallel multiple mediation is beneficial in comparing competing theories and determining which is the strongest mediator, as it provides specific indirect effects for each mediator while controlling for all other mediators by holding scores constant [36]. Conceptual diagrams for simple and parallel mediation models are provided in Fig. 1.

A similar step-down approach was used to examine the roles of depression, anxiety and stress. First, separate simple mediation analyses investigated the indirect effects of the three DASS subscales. If simple mediation analyses indicated significant indirect effects of more than one subscale, a multiple mediation analysis was conducted including depression, anxiety and stress as parallel mediators.

Finally, for any sleep parameters that had significant indirect effects of one or more DERS subscale *and* one or more DASS subscale in the simple mediation analyses outlined above, multiple mediation models (including all DERS and DASS subscales as parallel mediators) were analysed. Further, if the DERS total score *and* one or more DASS subscale displayed significant indirect effects in the simple mediation analyses, another multiple mediation analysis was conducted (including DERS total score and the three DASS subscales as parallel mediators). Pairwise comparisons between significant indirect effects were conducted for all multiple mediation models to determine whether effects significantly differed from one another.

## Results

PSQI, ISI, ESS and DASS data were collected and analysable from all participants. DERS data were not available for six participants in the BPD group. Actigraphy data were not analysable for four females (3 BPD, 1 healthy comparison); one due to a failure to return the actigraph, one due to dermal irritation, and two due to insufficient wear time (one and two days, respectively).

Final sample sizes were 78 (40 BPD, 38 HC) for subjective sleep and DASS analyses; 72 (34 BPC, 38 HC) for subjective sleep and DERS analyses; 74 (37 BPCD, 37 HC) for objective sleep and DASS analyses; and 68 (31 BPD, 37 HC) for objective sleep and DERS analyses.

Data regarding subjective chronotype (an individual’s natural inclination towards morning or evening preference for activity) is provided in Supplementary Material (see Table S1 and Fig. S1).

### Descriptive statistics and correlations

Group means and standard deviations for all variables and are displayed in Table 2. As this study was exploratory, independent samples t-tests (BPD vs. healthy comparison) were conducted for all variables, see Table 2.

**Table 2:** Mean (Standard Deviation) and Independent Sample t-test Results for Depression, Anxiety, Stress, Emotion Regulation, and Sleep Measures Across BPD and Healthy Compasison Groups

Measure	BPD	HC	Sig.
**Depression Anxiety Stress Scale**			
Depression	27.41 (11.18)	2.43 (3.47)	**
Anxiety	16.99 (7.98)	1.59 (2.22)	**
Stress	22.26 (9.73)	3.86 (3.45)	**
**Difficulties in Emotion Regulation Scale**			
Global score	127.73 (21.41)	63.79 (12.96)	**
Nonacceptance	18.97 (6.95)	9.74 (3.69)	**
Goals	20.18 (3.83)	12.57 (4.73)	**
Impulse	22.24 (5.76)	7.84 (1.59)	**
Aware	18.83 (6.07)	12.90 (3.46)	**
Strategies	30.49 (6.6)	12.10 (3.36)	**
Clarity	16.26 (4.52)	8.40 (2.04)	**
**Subjective Sleep**			
Pittsburgh Sleep Quality Index (PSQI)	10.41 (4.03)	5.27 (2.36)	**
Insomnia Severity Index (ISI)	14.76 (6.64)	4.04 (3.01)	**
Epworth Sleepiness Scale (ESS)	7.44 (4.46)	6.95 (4.31)	n.s.
**Objective Sleep**			
Bedtime (mins)	12:11 AM (89.91)	12:22 AM (58.92)	n.s.
Rise Time (mins)	9:20 AM (89.19)	8:18 AM (65.43)	*
Time in Bed (mins)	548.82 (79.95)	476.25 (43.17)	**
Total Sleep Time (mins)	492.50 (92.20)	431.05 (66.59)	*
Sleep Onset Latency (mins)	7.68 (7.26)	6.04 (8.19)	n.s.
Sleep Efficiency (%)	89.84% (9.98%)	90.24% (10.82%)	n.s.
Wake After Sleep Onset (mins)	45.14 (46.75)	36.16 (39.92)	n.s.
**Objective Sleep Variability**			
Bedtime (SD-mins)	99.68 (55.28)	64.77 (39.84)	*
Rise Time (SD-mins)	98.68 (43.65)	67.89 (27.36)	**
Time in Bed (CV)	0.20 (0.11)	0.18 (0.09)	n.s.
Total Sleep Time (CV)	0.21 (0.10)	0.20 (0.10)	n.s.

*Note*. BPD Borderline personality disorder. *HC* Healthy comparison. *SD* Standard deviation. *CV* Coefficient of variation. ** = *p* < .001, * = *p* < .05, n.s. = non-significant (*p* > .05). *p* values represent results of independent samples t-tests between BPD and HC groups

Correlations between group, mediating and outcome variables are displayed in Table 3. Several outcome variables were not significantly correlated with group or any mediating variables and were therefore not included in the mediation analyses. These were: daytime sleepiness (ESS), bedtime, sleep onset latency, sleep efficiency, wake after sleep onset, time in bed variability and total sleep time variability. These variables also did not significantly differ between BPD and healthy comparison groups (see Table 2).

**Table 3:** Pearson and Point-Biserial Correlations Between Group, Mediating and Outcome Variables

	DASS			DERS						Group	
	Depression	Anxiety	Stress	Non-Accept	Goals	Impulse	Aware	Strategies	Clarity	DERS Total	0 = HC, 1 = BPD
**Insomnia Severity Index**	.723**	.776**	.745**	.512**	.562**	.735**	.486**	.758**	.633**	.725**	.720**
**Pittsburgh Sleep Quality Index**	.648**	.705**	.656**	.433**	.508**	.675**	.441**	.663**	.568**	.646**	.617**
**Epworth Sleepiness Scale**	.085	.172	.184	.093	.099	.066	.224	.061	.081	.111	.056
**Bedtime**	−.206	−.126	.021	−.030	− .030	− .072	− .166	− .114	−.167	− .103	− .072
**Rise Time**	.214	.282*	.369**	.365**	.259*	.294*	.309**	.290*	.258*	.351**	.372**
**Time in Bed**	.455**	.450**	.398**	.445**	.325**	.407**	.520**	.445**	.462**	.503**	.497**
**Total Sleep Time**	.383**	.354**	.308**	.297*	.267*	.307*	.364**	.380**	.362**	.386**	.261**
**Sleep Onset Latency**	−.025	−.009	−.014	.119	−0.003	.050	.022	−.009	.008	.051	.107
**Sleep Efficiency**	.048	.007	.006	−.063	.025	.010	−.054	.062	.016	.008	−.020
**Wake After Sleep Onset**	.015	.055	.057	.126	.014	.060	.156	−.001	.055	.069	.104
**Bedtime (SD)**	.351**	.442**	.426**	.211	.199	.303*	.035	.371**	.230	.297*	.345**
**Rise Time (SD)**	.331**	.351**	.343**	.266*	.315**	.279*	.044	.318**	.166	.308*	.394**
**Time in Bed (CV)**	.118	.158	.190	.123	.171	.049	−.038	.120	−.030	.086	.125
**Total Sleep Time (CV)**	.062	.112	.132	.136	.096	.007	−.020	.046	−.025	.045	.047
**Group (0 = HC, 1 = BPD)**	.833**	.795**	.782**	.648**	.666**	.869**	.524**	.874**	.755**	.880**	–

*Note.* All values represent Pearson correlation co-efficient, except for correlations involving Group where Point-Biserial correlations are presented instead given the categorical nature of the Group variable. *DASS* Depression Anxiety Stress Scale. *DERS* Difficulties in Emotion Regulation Scale. *HC* Healthy comparison. *BPD* Borderline personality disorder. *SD* Standard deviation. *CV* Coefficient of variation * *p* < .05. ** *p* < .01

### Subjective sleep

#### Emotion regulation

Simple mediation analyses indicated independent indirect effects of DERS total score, and impulse and strategies subscales in the relationship between group status and poor subjective sleep quality. Similarly, significant indirect effects of the impulse and strategies subscales were observed for subjective insomnia severity. See Table 4.

**Table 4:** Simple Mediation Analyses

DV	Simple Mediating Variable (M)	Effect of IV on M (a)	Effect of M on DV	Direct effect (c′)	Total effect (c)	Indirect effect	Indirect effect 95% BCa CI
			(b)			(a x b)	
**PSQI**	DERS-Total	63.95*	0.06*	1.41	5.13*	3.62*	[0.15, 6.53]*
	Non-Accept	9.23*	0.04	4.67*		0.36	[−1.53, 1.87]
	Goals	7.61*	0.13	4.00*		1.03	[−0.37, 2.40]
	Impulse	14.40*	0.30*	0.68		4.35*	[1.38, 6.58]*
	Awareness	5.93*	0.12	4.29*		0.74	[−0.36, 2.00]
	Strategies	18.39*	0.22*	0.97		4.06*	[1.14, 6.72]*
	Clarity	7.86*	0.2	3.44*		1.59	[−1.15, 3.90]
	Depression	24.97*	0.12*	2.02		3.11*	[0.71, 5.32]*
	Anxiety	15.40*	0.25*	1.22		3.92*	[1.85, 5.69]*
	Stress	18.40*	0.16*	2.17		2.97*	[0.65, 4.86]*
**ISI**	DERS-Total	63.95*	0.10*	3.92	10.71*	6.31	[−0.32, 11.45]
	Non-Accept	9.23*	0.1	9.27*		0.96	[−1.97, 3.49]
	Goals	7.61*	0.22	8.53*		1.7	[−0.44, 4.36]
	Impulse	14.40*	0.46*	3.54		6.69*	[2.02, 10.57]*
	Awareness	5.93*	0.22	8.96*		1.27	[−0.68, 3.42]
	Strategies	18.39*	0.43*	2.24		7.99*	[2.25, 12.83]*
	Clarity	7.86*	0.35	7.51*		2.72	[−1.95, 6.62]
	Depression	24.97*	0.20*	5.72*		4.99*	[0.28, 9.32]*
	Anxiety	15.40*	0.43*	4.17*		6.55*	[3.27, 9.45]*
	Stress	18.40*	0.30*	5.28*		5.43*	[1.80, 8.48]*
**Time in Bed**	DERS-Total	62.46*	0.56	38.47	73.48*	35	[−26.40, 92.22]
	Non-Accept	8.66*	2.3	53.81*		19.67	[−6.87, 48.20]
	Goals	7.40*	−0.07	73.99*		−0.51	[−24.43, 26.11]
	Impulse	14.10*	−0.99	87.43*		−13.96	[−91.20, 45.35]
	Awareness	6.08*	4.49*	46.18*		27.30*	[5.78, 55.36]*
	Strategies	18.08*	0.22	69.42*		4.06	[−67.85, 72.05]
	Clarity	7.69*	2.74	52.36*		21.12	[−25.86, 54.77]
**Bedtime Variability (SD)**	Depression	24.76*	0.71	17.43	34.91*	17.48	[−13.84, 59.95]
	Anxiety	15.05*	2.49*	−2.57		37.48*	[2.26, 74.47]*
	Stress	17.88*	1.75*	3.63		21.28	[−5.29, 72.64]

*Note.* The independent variable for all analyses was group status (0 = healthy, 1 = BPD). *PSQI* Pittsburgh Sleep Quality Index. *ISI* Insomnia Severity Index. *DV* Dependent variable. *IV* Independent variable. *M* Simple mediating variable. *SD* Standard deviation. *Significant, as indicated by confidence intervals not encompassing zero [36]

**Fig. 2:**
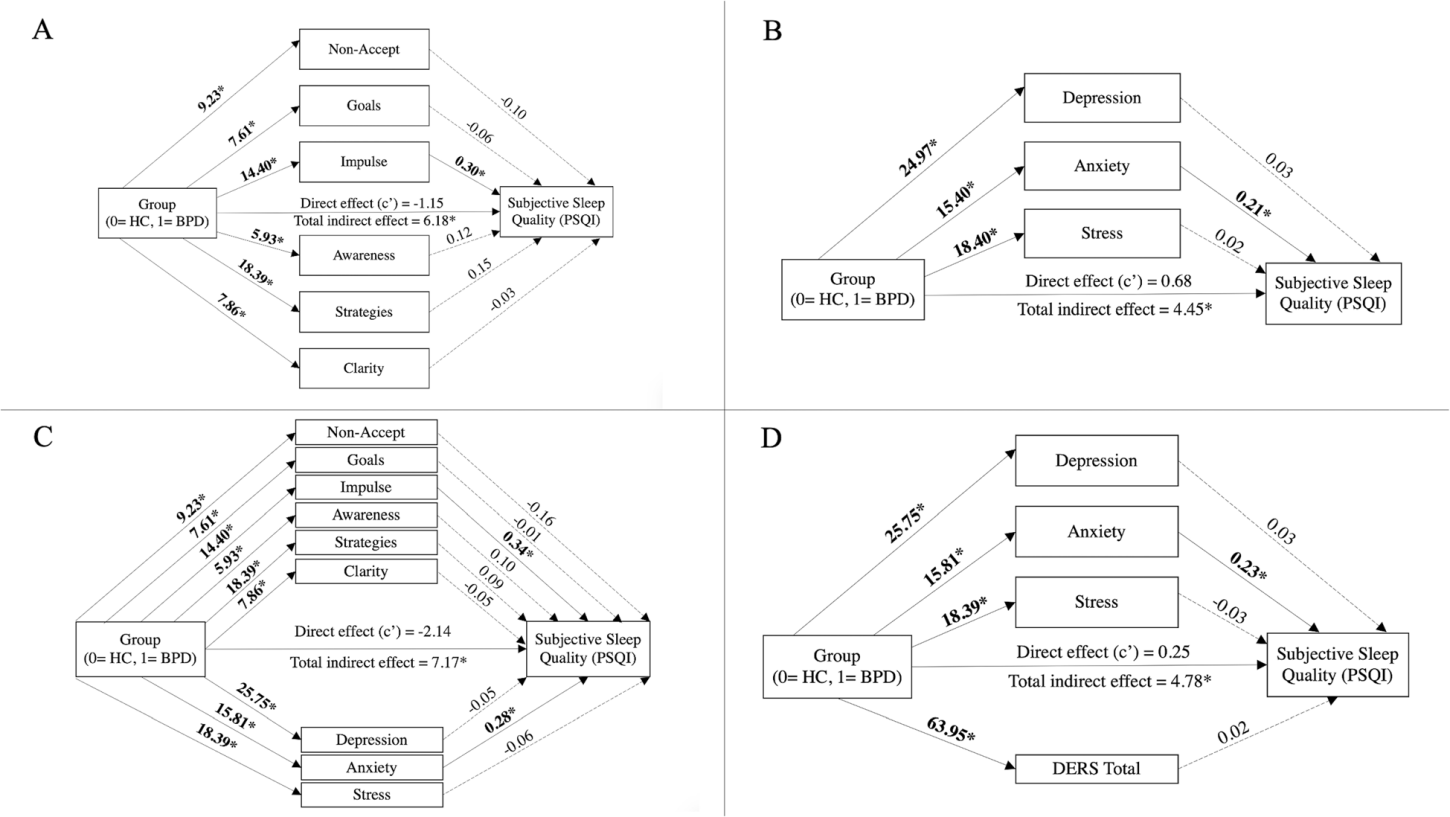
Indirect Roles of Emotion Regulation Difficulties, Depression, Anxiety and Stress in the Relationship Between Group Membership and Subjective Sleep Quality. *Note.* Solid lines and bolded values represent significant associations, based on 95% bias-corrected bootstrap 95% confidence intervals not including zero. Dashed lines represent non-significant associations. HC = healthy comparison. BPD = borderline personality disorder

Multiple mediation models including all DERS subscales as parallel mediators were analysed for subjective sleep quality and insomnia severity. For sleep quality, the overall indirect effect was significant (b = 6.18, 95% BCa CI [2.77, 8.97]), however only the indirect effect of the impulse subscale remained significant (b = 4.27, 95% BCa CI [1.07, 7.58]), see Fig. 2A.

**Fig. 3:**
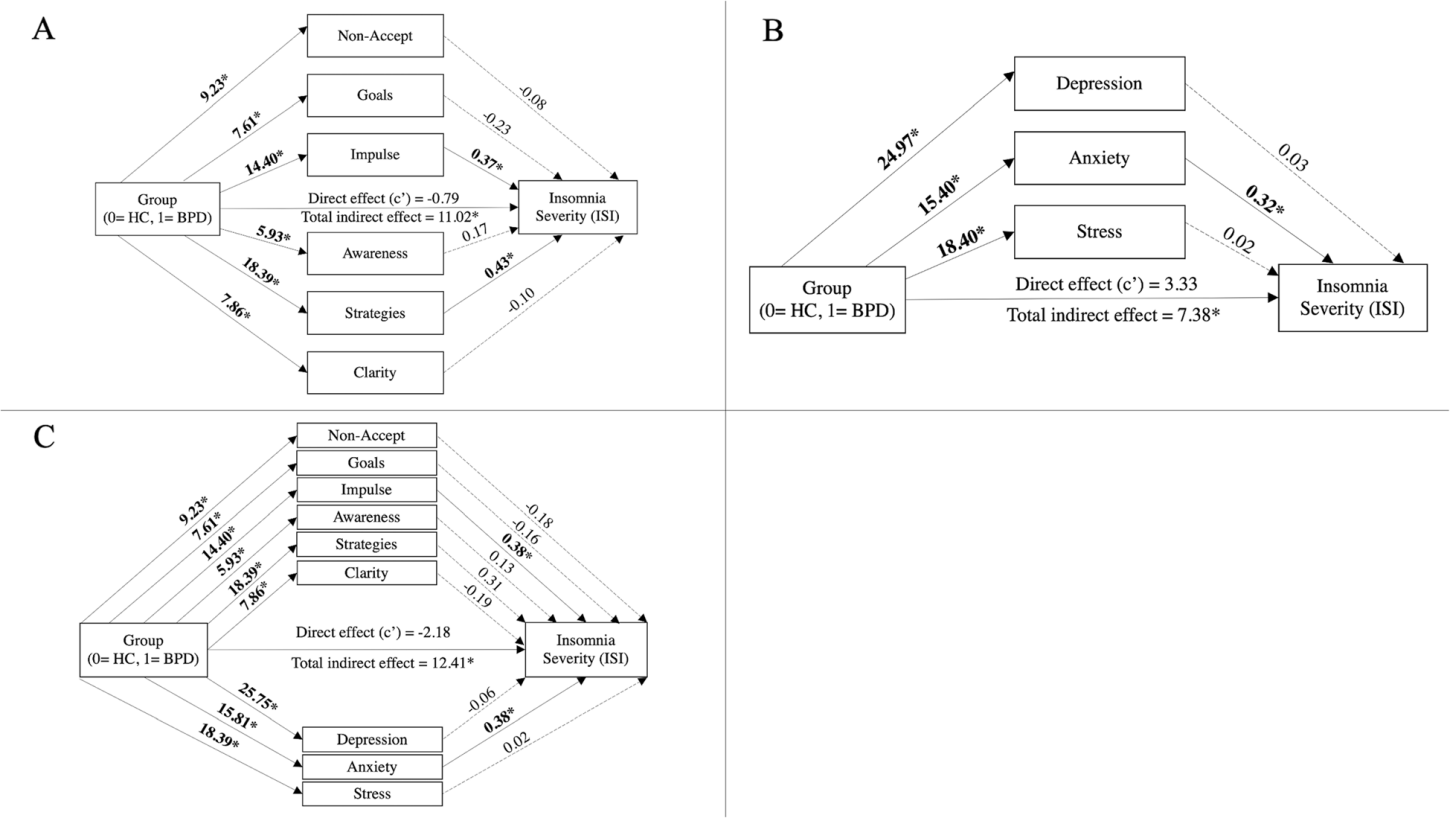
Indirect Roles of Emotion Regulation Difficulties, Depression, Anxiety and Stress in the Relationship Between Group Membership and Subjective Insomnia Severity. *Note.* Solid lines and bolded values represent significant associations, based on 95% bias-corrected confidence intervals not including zero. Dashed lines represent non-significant associations. HC = healthy comparison. BPD = borderline personality disorder

The overall indirect effect for insomnia severity was also significant (b = 11.02, 95% BCa CI [4.59, 16.33]), whilst the indirect effects of the impulse (b = 5.30, 95% BCa CI [0.27, 10.16]) and strategies (b = 8.00, 95% BCa CI [0.27, 15.70]) subscales both remained significant, see Fig. 3A. Pairwise comparisons revealed that the indirect effects of the impulse and strategies subscales were not significantly different (b = − 2.69, 95% BCa CI [− 13.00, 7.53]).

#### Depression, anxiety and stress

Simple mediation analyses revealed independent indirect effects of depression, anxiety and stress, in the relationships between BPD and subjective sleep quality and insomnia (Table 4). Multiple mediation models including depression, anxiety and stress as parallel mediators were analysed for subjective sleep quality and insomnia severity. For subjective sleep quality the overall indirect effect was significant (b = 4.45, 95% BCa CI [1.86, 6.59]), however only the indirect effect of anxiety remained significant (b = 3.23, 95% BCa CI [0.15, 5.97]) (Fig. 2B).

Similarly, the overall indirect effect for insomnia was significant (b = 7.38, 95% BCa CI [2.54, 11.41]), and only anxiety displayed a significant effect (b = 4.92, 95% BCa CI [0.38, 9.60]) (Fig. 3B).

#### Emotional regulation, depression, anxiety, and stress

Multiple mediation models, with all DERS and DASS subscales included as parallel mediators, were analysed for subjective sleep quality and insomnia severity. A second multiple mediation model, including DERS total, depression, anxiety, stress as parallel mediators, was analysed for subjective sleep quality.

Regarding sleep quality, the first multiple mediation model revealed a significant total indirect effect (b = 7.17, 95% BCa CI [3.80, 9.95]), and the indirect effects of the impulse (b = 0.58, 95% BCa CI [1.32, 8.37]) and anxiety (b = 4.43, 95% BCa CI [0.53, 7.74]) subscales remained significant (Fig. 2C). Pairwise comparisons revealed that the indirect effects of the impulse and anxiety subscales were not significantly different (b = 0.11, 95% BCa CI [− 0.93, 1.15]).

The second multiple mediation model indicated a significant total indirect effect (b = 4.78, 95% BCa CI [1.27, 7.64]), however only the indirect effect of anxiety remained significant (b = 3.64, 95% BCa CI [0.33, 6.69]) (Fig. 2D).

Regarding insomnia severity, the multiple mediation model revealed a significant total indirect effect (b = 12.41, 95% BCa CI [6.09, 17.66]).The indirect effects of the impulse (b = 5.44, 95% BCa CI [0.13, 10.95]) and anxiety (b = 5.98, 95% BCa CI [0.69, 10.69]) subscales remained significant (Fig. 3C). Pairwise comparisons revealed that the indirect effects of the impulse and anxiety subscales were not significantly different (b = − 0.55, 95% BCa CI [− 7.22, 6.39]).

### Objective sleep

#### Emotion regulation

Table 4 shows that there was an indirect effect of the awareness subscale in the relationship between BPD features and time in bed. No indirect effects of emotion regulation difficulties were observed for the remaining objective sleep parameters.

#### Depression, anxiety and stress

There was an indirect effect of anxiety in the relationship between BPD features and bedtime variability (Table 4). No indirect effects of depression, anxiety or stress were observed for the remaining objective sleep parameters.

## Discussion

This exploratory study aimed to investigate potential underlying mechanisms to inform future hypotheses and research development. It explored the roles of emotional dysregulation, depression, anxiety and stress in the relationship between BPD features and sleep disturbance in young people. Global difficulties in emotional regulation, impulse control difficulties, limited access to emotion regulation strategies, depression, anxiety and stress all indirectly contributed to the relationship between BPD features and subjective sleep disturbance. Further, lack of emotional awareness and anxiety contributed to the associations between BPD features and objectively longer time in bed and bedtime variability, respectively.

### Subjective sleep

The current findings align with extant research indicating that subjective sleep disturbance is associated with emotional dysregulation [27, 61], maladaptive coping strategies [35], impulsivity [5, 62, 73], depression, anxiety, and stress [2, 57, 64]. Notably, global emotion regulation difficulties contributed to poor global sleep quality, but not to insomnia severity specifically. This might help to explain inconsistencies in previous research, where one study used a general measure of sleep quality [29] while another focussed on more specific aspects of sleep such as sleep/wake behaviour problems [75].

The findings partially supported previous research. However, the effect of impulse control difficulties was unexpected [29]. Impulse control difficulties increase with BPD severity [16] and Grove et al.’s [29] student sample had less severe BPD pathology than is typically observed in clinical populations. This might help to explain why an effect of impulse control difficulties was observed in the current study, but not in Grove et al. [29]. Moreover, no effects of the DERS nonacceptance and clarity subscales were observed, contradicting previous reports [29]. Again, it is possible that differences in BPD severity played a role in these discrepant findings. However, as disorder severity was not assessed in the current study, this suggestion remains speculative and warrants further investigation.

The key mechanisms underlying subjective sleep disturbance in young people with BPD features were impulse control difficulties, limited access to emotion regulation strategies, and anxiety. These factors are interrelated [65, 66] but are independently associated with heightened arousal [20, 49, 67, 76, 77]. Cognitive and physiological arousal exacerbate subjective sleep disturbance and individuals often attribute their sleep problems to uncontrollable cognitive arousal [33, 71]. Specifically, control-related cognitive activity, and an external locus of control, wherein individuals believe that their circumstances are due to external forces beyond their control, perpetuate sleep disturbance through fuelling sleep-related worry and arousal [31, 33, 70]. External locus of control and control-related thoughts are reflected in the impulse and strategies DERS subscales [37, 53]. For example, “I experience my emotions as overwhelming and out of control” (impulse) and “When I’m upset, I believe there is nothing I can do to make myself feel better” (strategies). Locus of control has been identified as a sleep treatment target in other mental disorders [17] and can be modified through cognitive behavioural intervention [74].

Impulse control difficulties might also be related to disturbed sleep through greater pre-sleep smartphone use in young people with BPD features. Smartphone dependence has been associated with impulse control difficulties (Chóliz, [18]), and pre-sleep smartphone use is known to disturb sleep quality and duration through increased physiological and cognitive arousal [32]. Impulse control difficulties in young people with BPD features might therefore result in greater difficulty switching off one’s smartphone to go to sleep. As the current study did not assess smartphone use, this suggestion remains speculative and warrants further investigation.

Overall, these preliminary findings suggest that addressing impulse control, emotion regulation strategies and anxiety through locus of control and pre-sleep arousal might be beneficial in improving subjective sleep in young people with BPD features. However, longitudinal data is needed to support these suggestions.

### Objective sleep

Emotional dysregulation, depression, anxiety or stress did not indirectly affect any objective sleep parameters other than longer time in bed and bedtime variability. This is likely because few objective sleep disturbances have been observed in young people with BPD features [38, 41, 42].

#### Lack of emotional awareness and longer time in bed

Lack of emotional awareness (eg. “I do [not] pay attention to how I feel”) might reflect a maladaptive coping strategy of emotional avoidance. Spending more time in bed can serve as a similar emotionally avoidant coping strategy [24, 39, 46], which might explain the relationship between these factors. Crucially, emotional avoidance perpetuates emotional distress [15] and sleep disturbance [63]. However, reducing time in bed can have considerable negative consequences, particularly in young people who are prone to insufficient sleep [10, 60]. Instead, focussing interventions on developing adaptive emotional coping strategies might be beneficial.

#### Anxiety and bedtime variability

The associations between BPD features, anxiety and bedtime variability were consistent with broader literature demonstrating a relationship between bedtime variability and anxiety in young people, due to day-to-day fluctuations in anxiety and arousal levels [7, 25]. Sleep timing consistency is as important as sleep duration for psychological well-being [25]. Anxiety might therefore be a beneficial treatment target to improve bedtime consistency in young people with BPD features. Again, longitudinal data will help to clarify these cross-sectional observations.

### Strengths 

This was the first study to utilise objective sleep measures in the exploration of mechanisms underlying sleep disturbance in young people with BPD features. The inclusion of a youth-specific, clinical sample allowed the findings to be representative of a ‘real world’ population of help-seeking young people with BPD features encountered in clinical settings. Further, the investigation of depression, anxiety and stress alongside emotional dysregulation was novel. The use of parallel multiple mediation techniques allowed a direct comparison of potential mediating factors [36]. The findings exposed the importance of considering anxiety in the field of sleep and BPD, which has been largely neglected in prior research [79].

### Limitations

The current study was limited by its small scale longitudinal nature . While the 10 day-study provided valid sleep parameters and insights into intra-individual sleep variability, it was limited in assessing the temporal ordering or causal relationships between BPD features, emotion dysregulation, depression, anxiety, stress, and sleep disturbance in young people. Additionally, this study was limited in testing mediation [52]. Similar to Grove et al. [29], results from this study should be interpreted as being consistent with a mediational role in the relationship between BPD features and sleep disturbance, rather than mediation per se. Further, sleep disturbance and emotion dysregulation are bidirectionally related [27, 61], yet relationships explored in the current study were unidirectional. Future research should explore the bidirectional and longitudinal associations between BPD features, emotion dysregulation, depression, anxiety, stress, and sleep disturbance in young people. Additionally, examining the roles of BPD severity and medication use were beyond the scope of the current study and should be explored in future.

While actigraphy is a well-accepted and highly utilised method of objective sleep measurement, it cannot provide the same level of detailed sleep analysis as polysomnography and is limited by relatively poor specificity [1]. As such, objective sleep disturbances (eg. nighttime awakenings) might have been underestimated in the current study. Future research should examine the mechanisms underlying polysomnography-assessed sleep parameters in young people with BPD features.

## Conclusion

The current findings suggest that targeting impulse control, emotion regulation strategies and anxiety, through reducing external locus of control and pre-sleep arousal, might be beneficial in improving subjective sleep disturbance in young people with BPD features. Further, addressing emotional awareness and anxiety might be beneficial in further normalising objective sleep patterns in this population. These findings provide helpful guidance for future research hypotheses and the development of targeted sleep-improvement strategies that might improve current interventions for young people with BPD features.

## Supplementary Information

**Additional file 1 **Indirect role of emotion regulation difficulties, depression, anxiety and stress in the relationship between group membership and chronotype (an individual’s natural inclination towards morning or evening preference for activity).

## Data Availability

The datasets generated and/or analysed during the current study are not publicly available but are available from the corresponding author on reasonable request.
