# Supplementary material for: Potential mechanisms underlying sleep disturbance in young people with borderline personality disorder features: an exploratory study
Source: Borderline Personal Disord Emot Dysregul. 2022 Mar 10;9:10. doi: 10.1186/s40479-022-00180-2 (PMC8908552; doi:10.1186/s40479-022-00180-2)
Supplement: Supplementary file 1 — Additional file 1 Indirect role of emotion regulation difficulties, depression, anxiety and stress in the relationship between group membership and chronotype (an individual’s natural inclination towards morning or evening preference for activity). [file 40479_2022_180_MOESM1_ESM.docx]

**Supplementary Material**

**Chronotype Data**

Simple mediation analyses revealed that non-acceptance of emotions, impulse control difficulties and stress had significant indirect effects in the relationship between group status (BPD features vs. healthy) and later chronotype (as assessed using the Munich Chronotype Questionnaire: MCTQ), see Table S1.

**Table S1**

*Simple Mediation Analyses*

| **Dependent Variable** | **Simple Mediating Variable (M)** | **Effect of IV on M (a)** | **Effect of M on DV  (b)** | **Direct effect (c′)** | **Total effect (c)** | **Indirect effect  (a x b)** | **Indirect effect 95% BCa CI** |
| --- | --- | --- | --- | --- | --- | --- | --- |
| MCTQ | DERS-Total | 62.11* | 1.02 | -14.37 | 49.01* | 63.39 | [-4.41, 141.42] |
|  | Non-Accept | 9.70* | 4.59* | 4.52 |  | 44.49* | [2.66, 88.20]* |
|  | Goals | 6.81* | 1.84 | 36.46 |  | 12.56 | [-20.74, 45.84] |
|  | Impulse | 14.45* | 5.74* | -33.92 |  | 82.94* | [8.98, 173.18]* |
|  | Awareness | 6.20* | 2.40 | 34.17 |  | 14.85 | [-5.82, 47.79] |
|  | Strategies | 16.72* | 2.37 | 9.40 |  | 39.62 | [-16.77, 100.96] |
|  | Clarity | 7.65* | -0.95 | 56.25 |  | -7.23 | [-55.19, 36.43] |
|  | Depression | 25.33* | 0.32 | 53.93 | 62.06* | 8.13 | [-62.15, 65.68] |
|  | Anxiety | 15.24* | 1.38 | 41.03 |  | 21.03 | [-36.77, 67.49] |
|  | Stress | 18.16* | 3.72* | -5.41 |  | 67.47* | [13.22, 123.55]* |

*Note.* MCTQ = Munich Chronotype Questionnaire.

As multiple emotional dysregulation domains displayed significant indirect effects in the relationship between BPD and chronotype, multiple mediation analyses were conducted including all six emotional dysregulation domains as parallel mediators. The overall positive indirect effect was significant (*b* = 98.08, 95% BCa CI [25.67, 207.58]), as was the indirect effect of the nonacceptance subscale (*b* = 64.39, 95% BCa CI [7.19, 109.53]). Note that paths a and b were both significant for the impulse and clarity scales, however the indirect effects of these subscales were not significant, (*b* = 105.38, 95% BCa CI [7.19, 109.53] for impulse, *b* = -77.54, 95% BCa CI [-138.12, 1.35] for clarity). See Figure S1A.

Multiple mediation analyses were also conducted including all six emotional dysregulation domains and depression, anxiety and stress as parallel mediators. The overall positive indirect effect was significant (*b* = 98.84, 95% BCa CI [12.01, 212.84]), as were the indirect effects of nonacceptance (*b* = 62.44, 95% BCa CI [7.84, 110.48]), goals (*b* = -39.78, 95% BCa CI [-89.88, -3.74]), clarity (*b* =-86.28, 95% BCa CI [-154.56, -11.59]), and stress (*b* = 132.74, 95% BCa CI [5.67, 255.61]). Note that paths a and b were both significant for the impulse subscale, however the indirect effect was not significant, (*b* = 108.29, 95% BCa CI [-23.94, 201.20]). See Figure S1B.

**Figure S1**

*Indirect Role of Emotion Regulation Difficulties, Depression, Anxiety and Stress in the Relationship Between Group Membership and Chronotype
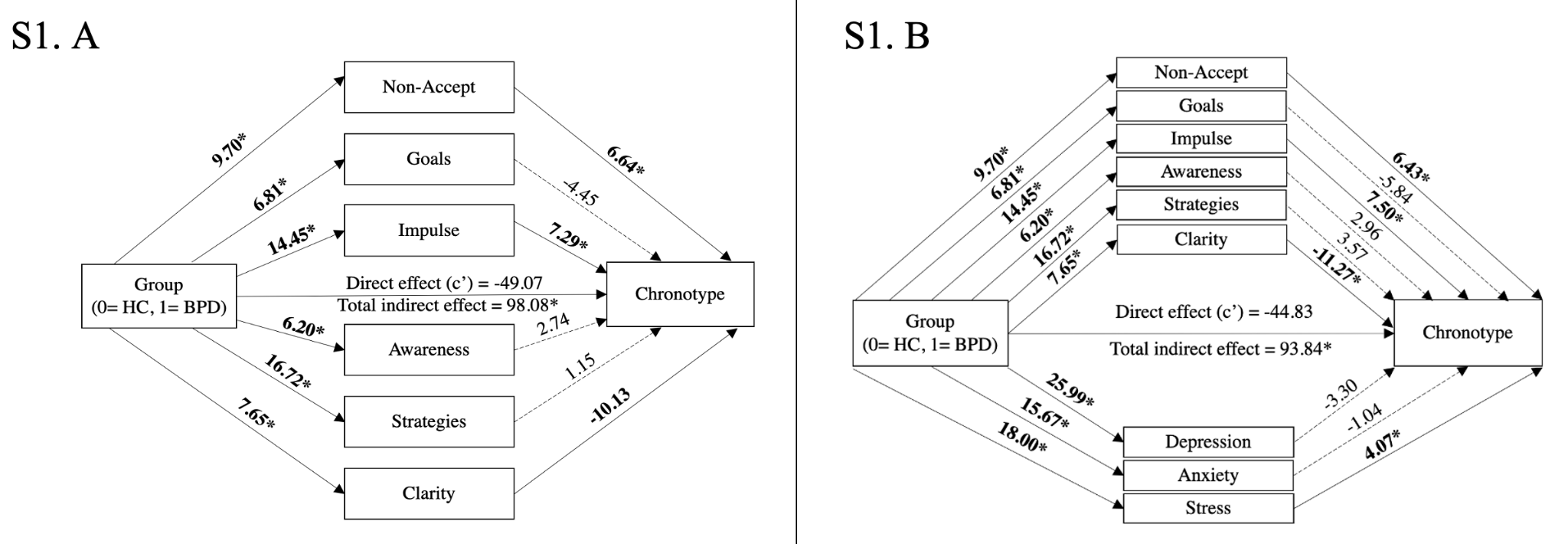
*

*Note.* Solid lines and bolded values represent significant associations, based on 95% bias-corrected confidence intervals not including zero. Dashed lines represent non-significant associations. HC= healthy comparison. BPD = borderline personality disorder.
